# Prenatal exposure to gestational diabetes mellitus increases developmental defects in the enamel of offspring

**DOI:** 10.1371/journal.pone.0211771

**Published:** 2019-02-27

**Authors:** Tawana Pascon, Angélica M. P. Barbosa, Rita C. L. Cordeiro, Diego G. Bussaneli, Caroline B. Prudencio, Sthefanie K. Nunes, Fabiane A. Pinheiro, Grasiela Bossolan, Leandro G. Oliveira, Iracema M. P. Calderon, Gabriela Marini, Marilza V. C. Rudge

**Affiliations:** 1 Department of Gynecology and Obstetrics, São Paulo State University (UNESP), Botucatu Medical School, Botucatu, São Paulo, Brazil; 2 Department of Physiotherapy and Occupational Therapy, São Paulo State University (UNESP), School of Philosophy and Sciences, Marília, São Paulo, Brazil; 3 Department of Pediatric Dentistry and Orthodontics, São Paulo State University (UNESP), Araraquara School of Dentistry, Araraquara, São Paulo, Brazil; 4 Health Sciences Center, University of the Sacred Heart (USC), Bauru, São Paulo, Brazil; Virgen Macarena University Hospital, School of Medicine, University of Seville, SPAIN

## Abstract

**Background and objective:**

Gestational diabetes mellitus (GDM) is associated with short- and long-term maternal and perinatal repercussions. Our objective was to evaluate the long-term consequences of intrauterine exposure to hyperglycemia on Developmental Defects of Enamel (DDE) in offspring.

**Results:**

Overall, 50 children of women with GDM and 250 children of normoglycemic women participated, the latter serving as controls. Children were examined at the age between 3 and 12 years. In addition to physical examination, two independent observers examined and rated photographs to identify specific types of DDE in a blinded fashion. Among offspring of mothers with GDM, rates of DDE (all types combined) and hypoplasia (specific type) were significantly higher (p<0.001, p = 0.04), in comparison to offspring of normoglycemic mothers. Considering only the affected teeth (1060 in GDM category; 5499 in controls), rates of DDE (all types combined) were significantly higher for total teeth (p <0.001) and deciduous teeth (p<0.001), but not permanent teeth. In specific types of DDE involving deciduous teeth, rates of demarcate opacity were significantly higher (p<0.001; canine and 2nd mandibular molars) and hypoplasia (p <0.001; 2nd maxillary molars and 2nd mandibular molars). In permanent teeth, the rate of diffuse opacity in association with GDM was significantly higher (p<0.001; maxillary central incisors and 1st maxillary molars).

**Conclusion:**

GDM was associated with the adverse effects of DDE on offspring. This study lays the foundation for future studies to determine the impact of GDM on long-term risk of DDE.

## Introduction

Gestational diabetes mellitus (GDM) [1, 2] is associated with an increased risk of complications for both mother and baby during pregnancy as well as the postpartum period [3–6]. GDM is also associated with short- and long-term repercussions [7–11]. The effects of the diabetic intrauterine environment during gestation cannot be ignored and extend beyond those apparent at birth [12]. Currently, infant survival is the norm, but the long-term effects on the offspring of GDM mothers who are born today may differ from those reported many years ago.[13–16].

Several studies suggest that maternal health conditions, particularly hyperglycemia during pregnancy, can alter fetal development to affect organ formation and increase the risk of diseases [13–16] however, the effects of maternal diabetes on tooth development and the associated underlying mechanisms have not been thoroughly investigated [17].

Epidemiologic and animal model studies have shown that hyperglycemia changes the tooth development process by affecting tooth eruption and mineralization [18–20].

Developmental defects of enamel (DDE) may negatively affect oral health and aesthetics, cause tooth sensitivity, malocclusion [21] results in anesthesia difficulties because of the hypersensitivity [22]. Moreover, they are risk factors for caries lesions and erosion in children´s teeth [23]

The limited available studies focus on diabetes mellitus, and the animal studies have highly heterogeneous results, which are inconclusive. Besides, most of the original studies regarding the GDM mother´s offspring did not include data about DDE. We hypothesize that gestational dysglicemia may affect the enamel formation initiate intrauterine life from the 14^th^ to 32^nd^ gestational weeks [24, 25].

Thus, objective of this study was to evaluate the long-term consequences of intrauterine hyperglycemia exposure on DDE in offspring 3–12 years after birth. Specific analyses were performed to determine the DDE (all types combined) rate and specific type, the DDE-affected surface localization, and the dentition types, teeth groups and number of teeth affected according to the intrauterine chronology of dental enamel formation.

## Methods

### Setting and population

This study was part of a prospective cohort study to evaluate the short- and long-term effects of GDM on mothers and their offspring. The study was conducted in the Perinatal Diabetes Research Center (PDRC) of Botucatu Medical School/UNESP/ Brazil from March 2016 to September 2017. All mothers and offspring who visited the Perinatal Diabetes Research Center between 2003 and 2013 were invited to participate. The selection of mothers and children in the GDM group was performed at this center since it is a tertiary referral center for perinatal diabetes care.

### Sample size estimation

The sample size was calculated based on the estimated prevalence of 7% GDM [1] and 16% DDE [26], the absence of confounders, and the prevalence of type I errors = 0.20 and type II errors = 0.05. It was estimated that to detect differences greater or equal to 30%, 13 children were needed for the GDM mothers (GDM) group, and 184 children were needed for the normoglycemic mothers (NGT) control group.

### Selection of subjects

The children were included only if the mothers agreed to have a dental examination, and the mothers were informed that they could terminate the follow-up at any time. According to the Helsinki Declaration, written informed consent for the inclusion of their records was obtained from the mothers of all selected children.

**Fig 1:**
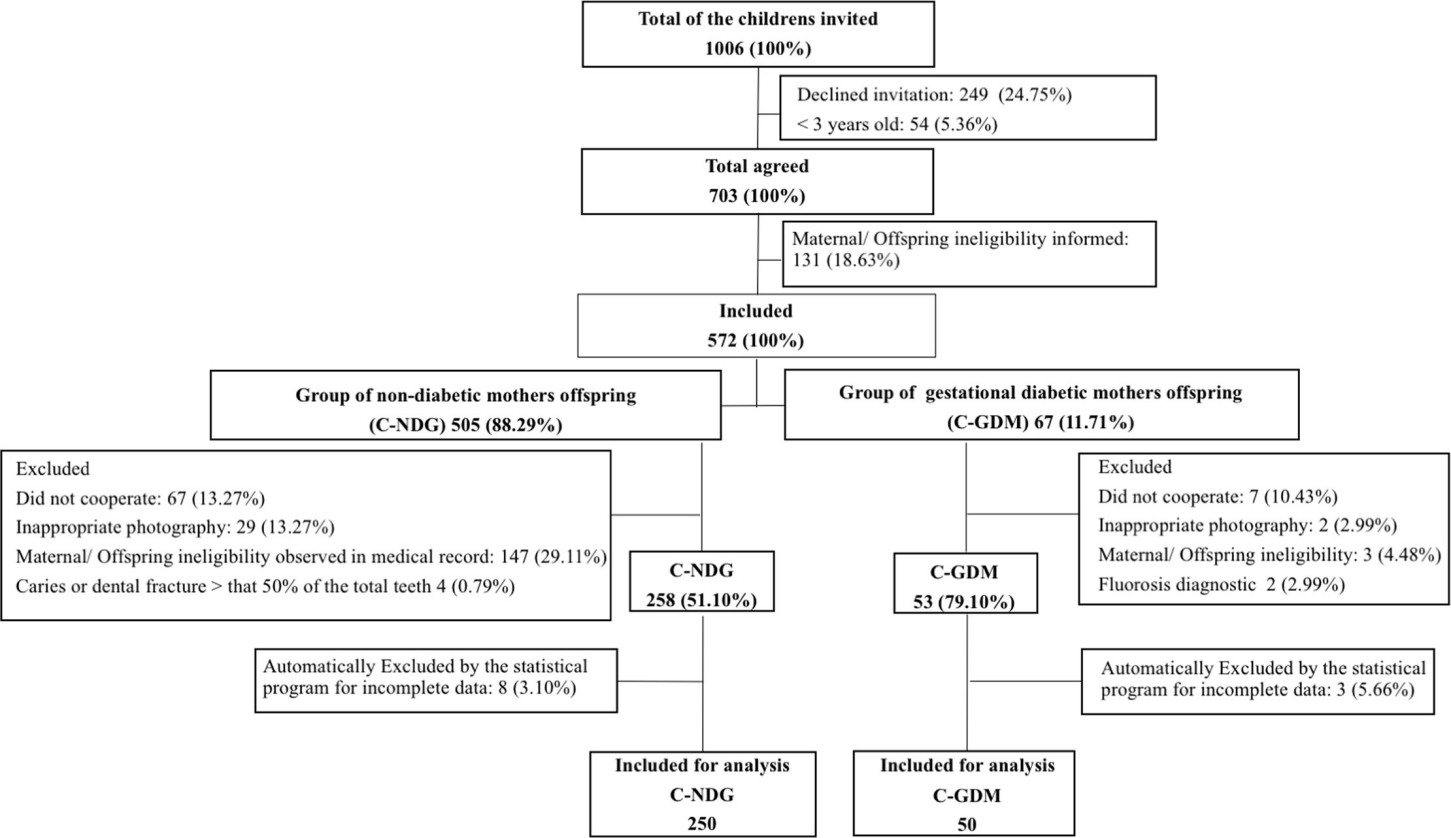
Flowchart of individuals enrolled in the study.

Access to the maternal and offspring data was approved by hospital trust administrations. Some of the mothers did not accept our invitation, and many children did not participate in the required clinical examination. Additionally, children with a history of systemic disease, deciduous tooth infection or trauma in the dentomaxillofacial region were excluded from the study. GDM was diagnosed with a 75-g glucose tolerance test (75-g GTT) as recommended by the American Diabetes Association[1], and the glucose profile test was performed as recommended by Rudge [3] between the 24th and 28th gestational weeks. All mothers with GDM received glucose-lowering treatment consisting of dietary and lifestyle counseling, and no mothers needed insulin therapy during pregnancy. The criteria for maternal and offspring ineligibility [24, 27–35] were defined based on all variables clearly mentioned in the literature as possible risk factors for the development of GDM. The maternal inclusion criteria were women with a GDM or NGT diagnosis [1, 3] and whose offspring were three to 12 years old. The mothers and their children who accepted the invitation (n = 572) were included as participants. Children were classified according to intrauterine hyperglycemia exposure (GDM study group, n = 50) or intrauterine normoglycemic exposure (NGT control group, n = 250) (Fig 1).

### Information collection

A structured medical history questionnaire was sent to the subjects. Background information solicited from the mother included gestational history, birth history, neonatal history and infant history and was obtained in the form of self-reported questionnaires, medical records and dental records. After their parents signed the informed consent form, the children were examined at the ambulatory clinic according to the World Health Organization guidelines for epidemiological studies on oral health. All data were entered in the PDRC electronic database of Botucatu Medical School.

### Examiner standardization

Prior to the examinations, a calibration exercise [36] using 41 clinical photographs of patients obtained from the Department of Pediatric Dentistry at Araraquara Dental School was conducted for the two examiners. A month after this exercise, the clinical photographs were re-examined by the same two examiners, and the kappa statistics [37] were used to measure the concordance between the examiners. The intra-examiner test agreement for DDE was excellent (0.79 and 0.82), inter-examiner good and excellent (0.67 and 0.75) and gold standard excellent (0.69 and 0.76; 0.76 and 0.79) reliability [36, 37].

### Dental clinical examination and DDE diagnostic criteria

The examinations were performed using a headlight, a plane intra-oral disposable mouth mirror, clinical instruments, an infant C-shaped labial dental retractor and sterile gauze for teeth cleaning and drying. The intraoral examinations were performed using biosafety material and protection for the examiner and child. Prior to the examinations, the children's teeth were cleaned and dried with gauze to remove any gross plaque or food deposits that may have been present [35].

The total number of existing teeth were counted and identified as deciduous or permanent teeth. The examinations for diagnosing DDE (all types comibined) and the specific type DDE for all teeth surfaces were performed in accordance with FDI criteria (FDI) [25] for the Defects of Dental Enamel Development (DDE) Index.

A probe was used to detect and confirm the presence of any tooth enamel surface discontinuity, and the child was examined in a sitting position on a chair or lying on a stretcher. DDE and the type of DDE were established by clinical examination, and the clinical diagnosis was confirmed by two blinded examiners by using teeth photographs [38]. Occlusal photographs were taken from the upper and lower arches, as well as lateral photographs on both sides and frontal photographs, using a digital camera (Sony Cyber-Shot, 162 megapixels Exnos R, 30x optical zoom, DSC HXICOV—HDAVCHD).

The diagnostic criteria for the occurrence of DDE and its three types (modified DDE index) were standardized for both the clinical examiner and the examiners who confirmed the photographs [24]. The three main types of enamel defects based on macroscopic appearance, namely, demarcated opacity, diffuse opacity and hypoplasia, were defined as the types of DDE. Fluorosis was evaluated [39] only to differentiate it from DDE and was excluded from the analysis [34, 36, 40].

### Ethics statement

Ethical permission was obtained from the Institutional Ethical Committee of Botucatu Medical School of São Paulo State University (CAAE 60537316.3.0000.5411).

### Statistical methods

Statistical analyses were performed using SPSS v21.0 (IBM, Armonk, NY, USA). Demographic data were reported as the mean and standard deviation or as percentages for categorical variables. Descriptive data analyses and odds ratios with 95% confidence intervals (CI's) were estimated. Non-normally distributed variables were analyzed using non-parametric tests; chi-square and Fisher’s exact tests were used to compare qualitative demographic data. To assess the relative strength of the association between the occurrence of GDM and DDE and various potential risk factors unadjusted logistic regression analyses were performed. The significance level was set at 0.05.

## Results

Table 1 compares demographic and anthropometric data among offspring of mothers with and without gestational diabetes mellitus, along with numbers of deciduous and permanent teeth evaluated. Among women with GDM, white ethnicity and overweight/obesity both occurred more frequently when compared to normoglycemic women.

**Table 1:** Demographic characteristics of the offspring of normoglycemic (NGT) and gestational diabetes mellitus (GDM) mothers, and of the number of deciduous and permanent teeth evaluated in the offspring in two groups.

Variable	NGT (n = 250)		GDM (n = 50)		*p* value
	Mean	SD	Mean	SD	
Age (year)	5.86	1.378	5.96	2.194	0.665
	n	%	n	%	
Gender					
Male	126	50.4	20	40.0	0.179
Female	124	44.6	30	60.0	0.179
Ethnicity					
White	99	39.6	33	66.0	0.001
Black	151	60.4	17	34.0	0.001
BMI (Kg/m^2^) at birth					<0.001
Normal weight	166	66.4	12	24.0	
Overweight/Obese	68	27.2	37	74.0	
Low weight	16	6.4	1	2.0	
Teeth evaluated (n = 6559)	21.23	1.893	21.20	2.000	0.915
Deciduous teeth evaluated (n = 5253)	17.07	3.061	16.66	4.289	0.425
Permanent teeth evaluated (n = 1306)	4.17	4.402	4.54	5.768	0.603

Chi-square or Fisher's exact tests. Statistically significant *p* values were less than 0.05. Body mass index (BMI).

The analysis of the occurrence or not of DDE (all types combined) showed that of the 250 offspring analyzed in the NGT group, 229 (91.6%) did not present DDE and 25 (10%) presented some specific type of DDE, and of the 50 offspring of the GDM group, 37 (64%) did not present DDE and 13 (26%) presented some type of DDE. We observed that there was no association between hypoplasia or even between opacities in the same offspring. We observed that the same opacity occurred in the deciduous and permanent dentition of the same offspring.

Table 2 compares the frequency distribution of DDE (all types combined) and specific type of DDE among offspring of mothers with and without GDM. Rates of DDE and hypoplasia were both significantly higher in the presence of GDM. Differences were not found between groups for demarcate opacity and diffuse opacity. There was no association between hypoplasia or even between opacities in the same offspring. There was only concomitance of opacity in the dentition of the same offspring. Only two cases of fluorosis were found in the GDM group, and none were found in the normoglycemic group; thus, fluorosis was excluded from the analysis.

**Table 2:** Comparison of the frequency of developmental defects of enamel (DDE) and specific type of DDE between the offspring of normoglycemic (NGT) and gestational diabetes mellitus (GDM) mothers.

Variable	NGT (n = 250)		GDM (n = 50)		*p* value
	n	%	n	%	
DDE	25	10.0	13	26.0	<0.001
Type DDE					
Demarcate Opacity	5	2.0	3	6.0	0.132
Diffuse Opacity	2	0.8	2	4.0	0.131
Hypoplasia	18	7.2	8	16.0	0.043

Chi-square or Fisher's exact test. Statistically significant p values were less than 0.05.

Table 3 shows, by the adjusted logistic regression model, that DDE (all types combined) and demarcate opacity (independent variables) were associated with GDM. No association was found between diffuse opacity or hypoplasia and GDM.

**Table 3:** Adjusted logistic regression model for association of the gestational diabetes mellitus (GDM) with developmental defects of enamel (DDE) and specific type of DDE in offspring dentition.

Variable	OR	95% CI	*p* value
DDE	3.04	1.34–6.92	<0.001
Type DDE			
Demarcate Opacity	12.54	1.73–90.87	0.012
Diffuse Opacity	4.49	0.53–37.76	0.166
Hypoplasia	1.88	0.73–4.85	0.190

Logistic model adjusted for newborn gender, ethnicity and BMI. Statistically significant *p* values were less than 0.05.

**Fig 2:**
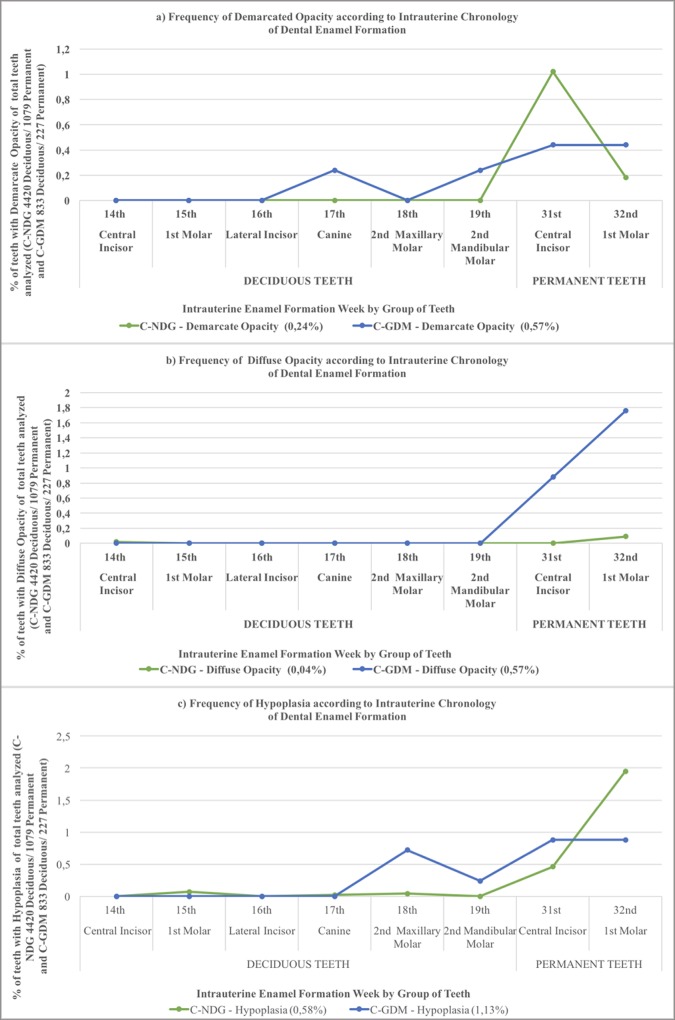
**Representation of the frequency of DDE according to the intrauterine chronology of dental enamel formation in groups of teeth and the following specific types of DDE in the teeth of the offspring of normoglycemic (NGT) and gestational diabetes mellitus (GDM) mothers: a) demarcate opacity, b) diffuse opacity and c) hypoplasia. Legend: Chi-square or Fisher's exact tests.** Statistically significant *p* values were less than 0.05.

Table 4 analyzes all permanent and deciduous teeth among offspring of mothers with and without GDM, while Fig 2 identifies specific teeth with DDE. Rates of DDE overall were significantly higher in the presence of GDM for total teeth and deciduous teeth, but not permanent teeth. Among specific DDE types, rates of demarcate opacity were significantly higher in deciduous teeth (canine and 2nd mandibular molars—Fig 2A)) and hypoplasia (2nd maxillary molars and 2nd mandibular molars–Fig 2C)). In permanent teeth, the rate of diffuse opacity in association with GDM was significantly higher (maxillary central incisors and 1st maxillary molars–Fig 2B)). In Fig 2, the groups of teeth with a higher rate of DDE in GDM offspring began forming enamel at the 17th week of intrauterine life, and this process continued throughout subsequent weeks.

**Table 4:** Rates of DDE in all teeth, deciduous teeth and permanent teeth and the types of DDE in all teeth, deciduous teeth and permanent teeth in the offspring of normoglycemic (NGT) and gestational diabetes mellitus (GDM) mothers.

Variable		NGT		GDM			*p* value
Total teeth evaluated		(n = 5499)		(n = 1060)			
		n	%	n	%		
(all teeth)	Demarcate Opacity	13	0.24	6	0.57	0.129	
Type DDE	Diffuse Opacity	2	0.04	6	0.57	<0.001	
	Hypoplasia	32	0.58	12	1.13	0.071	
Total Teeth with DDE		47	0.85	24	2.26	<0.001	
Total Deciduous teeth evaluated		(n = 4420)		(n = 833)			
		n	%	n	%		
Type DDE	Demarcate Opacity	0	0	4	0.5	<0.001	
	Diffuse Opacity	1	0.02	0	0	1.000	
	Hypoplasia	6	0.13	8	0.96	<0.001	
Total Deciduous Teeth with DDE		7	0.16	12	1.44	<0.001	
Total Permanent teeth evaluated		(n = 1079)		(n = 227)			
		n	%	n	%		
	Demarcate Opacity	13	1.20	2	0.88	1.000	
Type DDE	Diffuse Opacity	1	0.09	6	2.64	<0.001	
	Hypoplasia	26	2.40	4	1.76	0.727	
Total Permanent Teeth with DDE		40	3.71	12	5.29	0.357	

Chi-square or Fisher's exact tests. Statistically significant *p* values were less than 0.05.

## Discussion

Our findings showed higher rates of DDE and hypoplasia in the offspring of mothers with gestational diabetes. The logistic model adjusted for the gender, ethnicity and BMI of the newborns shows a higher risk of DDE and demarcated opacity in the offspring exposed to GDM.

In this study, the protocol, diagnostic criteria and classification of DDE were calibrated, and the literature indicates that this greatly improves the quality of the studies [41]. The diagnosis and classification of DDE were based on FDI criteria [25], and the frequency of the data was analyzed. The conclusion that the frequency of enamel defects is exclusive to the gestational phase is innovative; our study contributes greatly to resolving the conflict between studies examining risk factors for DDE because it establishes a methodology that reduces the diagnosis errors in these studies [33].

The prevalence of enamel defects (34.4%) found in our study for this population was similar to that found in other Brazilian studies (29.9%) [42, 43]. A diabetic intrauterine environment leads to increased susceptibility to disease in offspring [10, 44]; in our study, maternal disorders were related to enamel defects in 26% of GDM offspring and in 8.4% of control offspring. Our results were not in agreement with the literature, which reports rates of 40% and 70% [45–47]; the discrepancy is likely because these studies did not use the same ineligibility criteria as our study, which may have contributed to these lower rates. However, our findings highlight the importance of prenatal care in preventing maternal diseases and dental enamel disorders [45].

To analyze the prevalence of primary dentition enamel defects that began during pregnancy, longitudinal, descriptive and retrospective quantitative studies of children between 1 to 3 years of age were carried out in a city in the south of Brazil [19]. The predominant results showed that there were changes in the formation of primary teeth and in the appearance of opacities and hypoplasia; these symptoms were related to systemic complications during pregnancy [19]. Although a correlation has not been investigated, 3.2% of mothers have gestational diabetes; some animal [48] and human studies note that maternal systemic conditions, such as nutritional vitamin deficiencies of calcium and phosphorus and uncontrolled gestational diabetes, are possible factors that influence the prevalence of hypoplasia [19, 48].

Guapanchi et al. is one of the rare studies that has investigated the enamel defects of diabetic mothers; this study evaluated the occurrence of opacities beyond hypoplasia and observed a high correlation between gestational diabetes and enamel hypoplasia [8].

Our study also evaluated children between 3–12 years of age and found that 16% of patients in the GDM group had hypoplasia; this difference can be explained by the fact that the Guapanchi et al. [8] study did not apply the same exclusion and ineligibility criteria.

The results found of the different types of DDE in the GDM group in our study, both in the deciduous and in the permanent dentition are partially similar to those of Guapanchi et al. [8] since we found hypoplasia in the deciduous 2^nd^ mandibular molars and maxillary molars and in the permanent 1^st^ molars, maxillary canines and central incisors.

The negative impact in deciduous and permanent teeth may be impacted by due to the onset of glycemic change during pregnancy around the 18th to the 19th week of gestation, although the diagnosis is made weeks after this period. We demonstrated the possible influence of GDM from the first trimester, concomitant to the beginning of dental enamel formation (Fig 2). Our findings alert us to the need for diagnosis of GDM as soon as possible so that preventive and minimizing measures of deleterious effects can be adopted early [2].

Our findings are consistent with those of animal studies conducted by Afshar et al. [49] and Silva-Sousa et al.[19]; in a study conducted by Noren [50], there was no control group for comparison, but no relation was found between enamel hypoplasia and gestational diabetes [8]. These values cannot be compared because the percentage presented in the Guapanchi et al. [8] study was calculated according to the number of affected teeth, and in the present study, this percentage refers to the total number of teeth evaluated.

Our findings do not confirm the findings of other studies in which no associations between diabetes during gestation and defects in enamel development were observed. Such disagreements can be explained by the fact that the samples of these studies [21, 42, 47, 51] included 3.2% and 1% of mothers with gestational diabetes; in contrast, our study used a comparative group of mothers with gestational diabetes and another control group.

A study conducted in Hong Kong aimed to investigate the potential risk factors associated with the occurrence of DDE in primary teeth through a prospective cohort study using a random sample of children from the community. Diffuse opacities were the most common type of DDE. Several possible etiological factors were considered; however, after adjusting for confounding factors, no variable could be identified as a risk factor for DDE in this cohort study. When analyzing the studied variables, the authors did not identify whether there were mothers with a history of gestational diabetes or other well-established risk factors in this group [35].

In our group of children of diabetic mothers, we observed a difference in the occurrence of macrosomia, which is plausible because they are children of diabetic mothers. Although we have included this variable in the adjusted logistic regression analysis, in future studies, we must consider the findings of Wong et al. [52] who observed a significant association of the occurrence of demarcated opacities (p<0.05) for only the children with heavier birth weights.

In an experimental [53] study that used optical microscopy to analyze the enamel organ of the mandibular incisors of the offspring of rats with alloxan-induced diabetes, no alterations were found in the enamel organ of rats born to diabetic mothers in comparison with that of rats born to normal mothers. In contrast, significant differences were detected with computer-assisted morphometry. These results indicate that there are structural defects in the skeletal organ of rats born to mothers with alloxan-induced diabetes that can induce enamel hypoplasia that can be observed by scanning electron microscopy. The authors suggest that maternal metabolic changes are the cause of the hypoplasia. These findings support the hypoplasia results obtained in our study, but other defects should be evaluated in experimental studies using induced gestational diabetes models [54].

An experimental study that characterized the enamel hypoplasia in the offspring of rats with alloxan-induced diabetes mellitus led to research using pregnant rats supplemented or not with insulin and controls in which sterile saline was given instead of alloxan or insulin. The results showed that insulin treatment was not sufficient to prevent or reduce the occurrence of hypoplasia [19].

In the critical and delicate period of fetal development, the process by which a stimulus induces long-term impacts on the fetus, previously described and established as "fetal programming" by Hales and Barker [55], is a new concept called "metabolic memory". All metabolic abnormalities observed among gestational diabetic women create an in-utero environment for the fetus that programs for diseases during adulthood [53, 55]. This in-utero programming seems to create a kind of "metabolic memory” since the physiological anomalies of the gestational period are responsible for the onset of diseases when the offspring become adults [11, 53, 55].

DDE may impact the long term individual's health status, in both primary and permanent dentitions. The increased porosity of teeth affected by hypomineralization increases the risk of post-eruptive breakdowns and caries development [56] which can rapidly compromise tooth integrity, resulting in early dental loss. Although deciduous dentition is temporary, it is fundamental for craniofacial growth and development, mastication, speech and prevention of abnormal habits [57], and therefore, impacting the long-term health status. It is also noteworthy that when a DDE is detected in primary second molars, the chances of permanent teeth also being affected is greater, since the development period of the permanent molars and incisors coincides with that of the second primary molar [58].

Enamel development defects are directly related to hypocalcification, and the effects of gene alterations already demonstrated in different animal studies [59–62] should be investigated in GDM offspring to deepen our insight into the responses to this association. The chronological analysis indicates that the groups of teeth that had a higher rate of DDE in the GDM offspring began their enamel formation from the 17^th^ week of intrauterine life, and this development continued throughout subsequent weeks. A study comparing the cortical area of the humerus as measured by neonatal radiographs in children showed that children with enamel hypoplasia had a mean cortical area of 10.1±1.9 mm^2^ compared to 13.9±1.4 mm^2^ in children without enamel hypoplasia (p<0.001) [30]. These clinical findings of the positive relationship between demineralization due to probable hypocalcemia and enamel hypoplasia support the hypothesis that hypocalcemia in pregnancy complicated by diabetes [63, 64] is one of the causes of the prevalence of enamel development defects in the teeth of offspring [30, 65, 66].

This study is of paramount importance because dental enamel does not regenerate; it is a biological marker, and these defects are a reflection of in-utero events. Furthermore, because dental enamel defects can identify risk factors for health problems, they could auxiliary forensic identification, because enamel does not undergo remodeling and reabsorption. It is suggested that dental practitioners take note of these defects in routine practice and record maternal data so that dental record information is available during possible forensic investigations [67].

The strength of this study is that the knowledge of the distribution of enamel defects and the factors associated with their development allows a better understanding of the problem and its diagnosis; furthermore, this study can contribute to establishing measures of prevention and treatment of these defects

The American Diabetes Association (ADA) defines GDM as “Diabetes diagnosed in the second or third trimester of pregnancy that is not clearly overt diabetes”[1]; however, according to the International Association of Diabetes and Pregnancy Study Groups (IADPSG) criteria, women can be diagnosed with GDM in even the first trimester [2]. Thus, Fig 2 is a graphical representation of the frequency of teeth affected by different types of DDE for both NGT and diagnosed with GMD offspring, considering the period of dental formation in weeks of intrauterine life. From these findings, we can suggest the possible influence of the high glycemic index during the tooth enamel formation process. The findings of our study and the knowledge of the chronology of early enamel formation at the onset of intrauterine life reinforce the need to diagnose GDM to minimize the deleterious effects caused by the altered maternal environment.

Inevitably, the present study have limitations; some maternal and offspring variables, such as maternal blood data during gestation and follow-up dental eruption data in the offspring, would have given a more accurate assessment of GDM and the occurrence of DDE. This study is the first to perform clearly prospective and quantitative assessments of GDM and longitudinal measurements of the enamel of offspring teeth. Careful control of potential confounders has been considered to minimize the bias of reverse causality or unmeasured confusion.

To avoid bias in the results, model adjustments were made for gender because it precedes the occurrence of GDM and for ethnicity and BMI because they were different in the study group. We attempted to correct for as many of the confounding variables as possible in this study design; consequently, the design of this study is a robust alternative to assess the adverse effects on mothers and infants. The identification of non-modifiable risk factors that may influence the postnatal programming mechanisms of GDM in offspring is necessary to formulate DDE prevention strategies in this high-risk group. This study significantly advanced the current knowledge of the effects of GDM on DDE in offspring and suggests that susceptibility to DDE may be the result of the fetal programming induced by maternal diabetes.

This study also lays the foundation for future studies to determine the impact of GDM on the long-term risk of DDE. We believe that this study has important clinical relevance since it provides evidence of the connection between GDM and DDE and thus promotes interest in investigating preventive and therapeutic strategies for mothers and their children to avoid or minimize the consequences of GDM.

## Conclusion

In conclusion, in the present study, GDM was independently associated with the adverse effects of DDE on offspring, and the most common type of DDE that was associated with GDM was demarcated opacity. The prevalence of DDE was significantly higher in the offspring of GDM mothers, and the type of DDE with the highest rate of occurrence was hypoplasia. It was also concluded that there was a higher proportion of teeth with DDE in the offspring of GDM mothers; moreover, in the GDM group, the deciduous dentition had a significantly higher proportion of demarcated opacity and hypoplasia, and the permanent dentition had a significantly higher proportion of diffuse opacity. This study lays the foundation for future studies to determine the impact of GDM on long-term risk of DDE.

## Supporting information

S1 Fig Frequency of developmental defects of enamel (DDE) and type of DDE between the offspring of normoglycemic (NGT) and gestational diabetes mellitus (GDM) mothers. (PNG)

S2 Fig Frequency of teeth with developmental defects of enamel (DDE) of the offspring of normoglycemic (NGT) and gestational diabetes mellitus (GDM) mothers. (PNG)

## References

[1] Diagnosis and classification of diabetes mellitus. Diabetes Care. 2012;35 Suppl 1:S64–71. 10.2337/dc12-s06435/Supplement_1/S64 22187472 PMC3632174

[2] Weinert LS. International Association of Diabetes and Pregnancy Study Groups recommendations on the diagnosis and classification of hyperglycemia in pregnancy: comment to the International Association of Diabetes and Pregnancy Study Groups Consensus Panel. Diabetes Care. 2010;33(7):e97; author reply e8. 10.2337/dc10-0544 20587717

[3] Rudge M, Calderon I, Ramos M, Brasil M, Rugolo L, Bossolan G. Hiperglicemia materna diária diagnosticada pelo perfil glicêmico: um problema de saúde pública materno e perinatal. Rev Bras Ginecol e Obs. 2005;27(11).

[4] Buchanan TA, Xiang AH. Gestational diabetes mellitus. J Clin Invest. 2005;115(3):485–91. 10.1172/JCI24531 15765129 PMC1052018

[5] Maganha CA, Bernardini MA, Vanni DGBS, Nomura RMY, M. Z. Repercussões do diabetes no feto e recém-nascido. Rev Ginecol e Obstet. 1990;23(3):158–62.

[6] Meneses J, Diniz EM, Simoes F. V. Neonatal morbidity in neonates born to mothers with gestational diabetes.1999 Rev Pediatr.30–6.

[7] Catalano PM, McIntyre HD, Cruickshank JK, McCance DR, Dyer AR, Metzger BE, et al The hyperglycemia and adverse pregnancy outcome study: associations of GDM and obesity with pregnancy outcomes. Diabetes Care. 2012;35(4):780–6. Epub 10.2337/dc11-1790 22357187 PMC3308300

[8] Ghapanchi J, Kamali F, Siavash Z, Ebrahimi H, Pourshahidi S, Ranjbar Z. The Relationship between Gestational Diabetes, Enamel Hypoplasia and DMFT in Children: A Clinical Study in Southern Iran. British Journal of Medicine and Medical Research. 2015;10(9):1–6.

[9] Mitanchez D, Burguet A, Simeoni U. Infants born to mothers with gestational diabetes mellitus: mild neonatal effects, a long-term threat to global health. J Pediatr. 2014;164(3):445–50. 10.1016/j.jpeds.2013.10.076 24331686

[10] Wu CS, Nohr EA, Bech BH, Vestergaard M, Olsen J. Long-term health outcomes in children born to mothers with diabetes: a population-based cohort study. PLoS One. 2012;7(5):e36727 10.1371/journal.pone.0036727 22649497 PMC3359312

[11] Yessoufou A, Moutairou K. Maternal diabetes in pregnancy: early and long-term outcomes on the offspring and the concept of "metabolic memory". Exp Diabetes Res. 2011;2011:218598 10.1155/2011/218598 22144985 PMC3226356

[12] Dabelea D, Mayer-Davis EJ, Saydah S, Imperatore G, Linder B, Divers J, et al Prevalence of type 1 and type 2 diabetes among children and adolescents from 2001 to 2009. JAMA. 2014;311(17):1778–86. 10.1001/jama.2014.3201 24794371 PMC4368900

[13] Yogev Y, Visser GH. Obesity, gestational diabetes and pregnancy outcome. Semin Fetal Neonatal Med. 2009;14(2):77–84. 10.1016/j.siny.2008.09.002 18926784

[14] Wren C, Birrell G, Hawthorne G. Cardiovascular malformations in infants of diabetic mothers. Heart. 2003;89(10):1217–20. 12975424 10.1136/heart.89.10.1217 PMC1767924

[15] Wei D, Loeken MR. Increased DNA methyltransferase 3b (Dnmt3b)-mediated CpG island methylation stimulated by oxidative stress inhibits expression of a gene required for neural tube and neural crest development in diabetic pregnancy. Diabetes. 2014;63(10):3512–22. 10.2337/db14-0231 24834974 PMC4171658

[16] Vrachnis N, Antonakopoulos N, Iliodromiti Z, Dafopoulos K, Siristatidis C, Pappa KI, et al Impact of maternal diabetes on epigenetic modifications leading to diseases in the offspring. Exp Diabetes Res. 2012;2012:538474 10.1155/2012/538474 23227034 PMC3512252

[17] Chen G, Chen J, Yan Z, Li Z, Yu M, Guo W, et al Maternal diabetes modulates dental epithelial stem cells proliferation and self-renewal in offspring through apurinic/apyrimidinicendonuclease 1-mediated DNA methylation. Sci Rep. 2017;7:40762 10.1038/srep40762 28094306 PMC5240105

[18] Lal S, Cheng B, Kaplan S, Softness B, Greenberg E, Goland RS, et al Accelerated tooth eruption in children with diabetes mellitus. Pediatrics. 2008;121(5):e1139–43. 10.1542/peds.2007-1486 18450858

[19] Silva-Sousa YT, Peres LC, Foss MC. Enamel hypoplasia in a litter of rats with alloxan-induced diabetes mellitus. Braz Dent J. 2003;14(2):87–93. doi: S0103-64402003000200003 12964650 10.1590/s0103-64402003000200003

[20] Villarino ME, Goya JA, RC DEL, Ubios AM. Alterations of tooth eruption and growth in pups suckling from diabetic dams. Pediatr Res. 2005;58(4):695–9. 10.1203/01.PDR.0000180599.54807.24 . 16189195

[21] Hong L, Levy SM, Warren JJ, Broffitt B. Association between enamel hypoplasia and dental caries in primary second molars: a cohort study. Caries Res. 2009;43(5):345–53. 10.1159/000231571 19648745 PMC2814013

[22] Jalevik B, Noren JG. Enamel hypomineralization of permanent first molars: a morphological study and survey of possible aetiological factors. Int J Paediatr Dent. 2000;10(4):278–89. 11310241 10.1046/j.1365-263x.2000.00210.x

[23] Seow WK, Ford D, Kazoullis S, Newman B, Holcombe T. Comparison of enamel defects in the primary and permanent dentitions of children from a low-fluoride District in Australia. Pediatr Dent. 2011;33(3):207–12. 21703072

[24] Antoine D, Hillson S, Dean MC. The developmental clock of dental enamel: a test for the periodicity of prism cross-striations in modern humans and an evaluation of the most likely sources of error in histological studies of this kind. J Anat. 2009;214(1):45–55. 10.1111/j.1469-7580.2008.01010.x 19166472 PMC2667916

[25] A review of the developmental defects of enamel index (DDE Index). Commission on Oral Health, Research & Epidemiology. Report of an FDI Working Group. Int Dent J. 1992;42(6):411–26. 1286924

[26] Hoffmann HS, Sousa MLR, CYPRIANO S. Prevalência de defeitos de esmalte e sua relação com a cárie dentária nas dentições decídua e permanente, Indaiatuba, São Paulo, Brasil. Cad Saúde Pública. 2007;23(2). 10.1590/s0102-311x2007000200020 17221093

[27] Seow WK. Enamel hypoplasia in the primary dentition: a review. ASDC J Dent Child. 1991;58(6):441–52. Epub 1991/11/01. 1783694

[28] Seow WK, Brown JP, Tudehope DI, O'Callaghan M. Developmental defects in the primary dentition of low birth-weight infants: adverse effects of laryngoscopy and prolonged endotracheal intubation. Pediatr Dent. 1984;6(1):28–31. 6592545

[29] Seow WK, Humphrys C, Tudehope DI. Increased prevalence of developmental dental defects in low birth-weight, prematurely born children: a controlled study. Pediatr Dent. 1987;9(3):221–5. 3507638

[30] Seow WK, Masel JP, Weir C, Tudehope DI. Mineral deficiency in the pathogenesis of enamel hypoplasia in prematurely born, very low birthweight children. Pediatr Dent. 1989;11(4):297–302. 2639324

[31] Frias JL, Frias JP, Frias PA, Martinez-Frias ML. Infrequently studied congenital anomalies as clues to the diagnosis of maternal diabetes mellitus. Am J Med Genet A. 2007;143A(24):2904–9. 10.1002/ajmg.a.32071 18000913

[32] Standards of medical care in diabetes—2015: summary of revisions. Diabetes Care. 2015;38 Suppl:S4 10.2337/dc15-S00338/Supplement_1/S4 25537706

[33] Pinho J, Lamy Filho F, Thomaz É, Lamy Z, Cruz MN, Libério S. Prevalência de defeitos de desenvolvimento de esmalte na dentição decídua adquiridos na vida intrauterina. Rev Bras Odontol. 2011;68(1):118–23.

[34] World Health organization. Oral Health Surveys Basic Methods. 4th edn Geneva: WHO 1997.

[35] Wong HM, Peng SM, Wen YF, King NM, McGrath CP. Risk factors of developmental defects of enamel—a prospective cohort study. PLoS One. 2014;9(10):e109351 10.1371/journal.pone.0109351 25275499 PMC4183707

[36] Lin LI. A concordance correlation coefficient to evaluate reproducibility. Biometrics. 1989;45(1):255–68. 2720055

[37] McHugh ML. Interrater reliability: the kappa statistic. Biochem Med (Zagreb). 2012;22(3):276–82. 23092060 PMC3900052

[38] Fleiss I. Statistical Methods for Rates and Proportions, ed 2 New York, Wiley 1981:212–25.

[39] Sabokseir A, Golkari A, Sheiham A. Distinguishing between enamel fluorosis and other enamel defects in permanent teeth of children. PeerJ. 2016;4:e1745 10.7717/peerj.1745 26966672 PMC4782718

[40] Vello MA, Martinez-Costa C, Catala M, Fons J, Brines J, Guijarro-Martinez R. Prenatal and neonatal risk factors for the development of enamel defects in low birth weight children. Oral Dis. 2010;16(3):257–62. 10.1111/j.1601-0825.2009.01629.x 19849806

[41] Ghanim A, Silva MJ, Elfrink MEC, Lygidakis NA, Marino RJ, Weerheijm KL, et al Molar incisor hypomineralisation (MIH) training manual for clinical field surveys and practice. Eur Arch Paediatr Dent. 2017;18(4):225–42. 10.1007/s40368-017-0293-9 28721667

[42] Lunardelli SE, Peres MA. Prevalence and distribution of developmental enamel defects in the primary dentition of pre-school children. Braz Oral Res. 2005;19(2):144–9. doi: S1806-83242005000200013 16292449 10.1590/s1806-83242005000200013

[43] Oliveira AF, Chaves AM, Rosenblatt A. The influence of enamel defects on the development of early childhood caries in a population with low socioeconomic status: a longitudinal study. Caries Res. 2006;40(4):296–302. 10.1159/000093188 16741360

[44] Song Y, Niu T, Manson JE, Kwiatkowski DJ, Liu S. Are variants in the CAPN10 gene related to risk of type 2 diabetes? A quantitative assessment of population and family-based association studies. Am J Hum Genet. 2004;74(2):208–22. 10.1086/381400 14730479 PMC1181919

[45] Caixeta FF, Correa MS. [Evaluation of the dental eruption pattern and of enamel defects in the premature child]. Rev Assoc Med Bras (1992). 2005;51(4):195–9. doi: S0104-42302005000400014 16127578 10.1590/s0104-42302005000400014

[46] Dummer PM, Kingdon A, Kingdon R. Prevalence and distribution by tooth type and surface of developmental defects of dental enamel in a group of 15- to 16-year-old children in South Wales. Community Dent Health. 1990;7(4):369–77. 2292067

[47] Needleman HL, Allred E, Bellinger D, Leviton A, Rabinowitz M, Iverson K. Antecedents and correlates of hypoplastic enamel defects of primary incisors. Pediatr Dent. 1992;14(3):158–66. . 1528784

[48] Collodel A, Sonego FGF, Simoes PWTA Pires PDS, Ceretta RA, Ceretta LB, et al Análise da prevalência de defeitos de esmalte na dentição decíduav. REV ASSOC PAUL CIR DENT. 2015;69(4):412–20.

[49] Afshar H. Hypoplasia of deciduous teeth. J of Dentistry. 2002;14(1):25–33.

[50] Noren JG. Enamel structure in deciduous teeth from low-birth-weight infants. Acta Odontol Scand. 1983;41(6):355–62. 6581675 10.3109/00016358309162347

[51] Burguet A. Long-term outcome in children of mothers with gestational diabetes. Diabetes Metab. 2010;36(6 Pt 2):682–94. 10.1016/j.diabet.2010.11.018S1262-3636(10)00283-1 21163430

[52] Wong HM, McGrath C, King NM. Diffuse opacities in 12-year-old Hong Kong children—four cross-sectional surveys. Community Dent Oral Epidemiol. 2014;42(1):61–9. 10.1111/cdoe.12064 23889509

[53] Dörner G, Plagemann A. Perinatal hyperinsulinism as possible predisposing factor for diabetes mellitus, obesity and enhanced cardiovascular risk in later life. Hormone and Metabolic Research. 1994;26(4):213–21. 8076902 10.1055/s-2007-1001668

[54] Silva-Sousa YTC, Peres LC, Foss MC. Are there structural alterations in the enamel organ of offspring of rats with alloxan-induced diabetes mellitus? Brazilian Dental Journal. 2003;14(3):162–7. 15057390 10.1590/s0103-64402003000300004

[55] Hales CN, Barker DJ. The thrifty phenotype hypothesis. Br Med Bull. 2001;60:5–20. 11809615 10.1093/bmb/60.1.5

[56] Crombie FA, Manton DJ, Palamara JE, Zalizniak I, Cochrane NJ, Reynolds EC. Characterisation of developmentally hypomineralised human enamel. J Dent. 2013;41(7):611–8. Epub 2013/05/21. 10.1016/j.jdent.2013.05.002 S0300-5712(13)00125-5 [pii]. . 23685033

[57] Monte-Santo AS, Viana SVC, Moreira KMS, Imparato JCP, Mendes FM, Bonini G. Prevalence of early loss of primary molar and its impact in schoolchildren's quality of life. Int J Paediatr Dent. 2018;28(6):595–601. Epub 2018/08/15. 10.1111/ipd.12416 . 30105883

[58] Garot E, Denis A, Delbos Y, Manton D, Silva M, Rouas P. Are hypomineralised lesions on second primary molars (HSPM) a predictive sign of molar incisor hypomineralisation (MIH)? A systematic review and a meta-analysis. J Dent. 2018;72:8–13. Epub 2018/03/20. S0300-5712(18)30052-6 [pii] 10.1016/j.jdent.2018.03.005 . 29550493

[59] Yin K, Lei Y, Wen X, Lacruz RS, Soleimani M, Kurtz I, et al SLC26A Gene Family Participate in pH Regulation during Enamel Maturation. PLoS One. 2015;10(12):e0144703 10.1371/journal.pone.0144703 26671068 PMC4679777

[60] Wen X, Lacruz RS, Smith CE, Paine ML. Gene-expression profile and localization of Na+/K(+)-ATPase in rat enamel organ cells. Eur J Oral Sci. 2014;122(1):21–6. 10.1111/eos.12106 24313748 PMC4005357

[61] Hu P, Lacruz RS, Smith CE, Smith SM, Kurtz I, Paine ML. Expression of the sodium/calcium/potassium exchanger, NCKX4, in ameloblasts. Cells Tissues Organs. 2012;196(6):501–9. 10.1159/000337493 22677781 PMC3535175

[62] Bronckers AL, Lyaruu D, Jalali R, Medina JF, Zandieh-Doulabi B, DenBesten PK. Ameloblast Modulation and Transport of Cl(-), Na(+), and K(+) during Amelogenesis. J Dent Res. 2015;94(12):1740–7. 10.1177/0022034515606900 26403673 PMC4681480

[63] Amaral ACS, Andrade BP, Dias PFF, Fortuna RNI, Junior RMA, Tavares RT, et al Complicações neonatais do diabetes mellitus gestacional Rev Med Minas Gerais. 2012;22(Supl 5):S40–S2.

[64] Jones CW. Gestational diabetes and its impact on the neonate. Neonatal Netw. 2001;20(6):17–23. Epub 2002/07/30. 10.1891/0730-0832.20.6.17 . 12144115

[65] Asemi Z, Karamali M, Esmaillzadeh A. Effects of calcium-vitamin D co-supplementation on glycaemic control, inflammation and oxidative stress in gestational diabetes: a randomised placebo-controlled trial. Diabetologia. 2014;57(9):1798–806. 10.1007/s00125-014-3293-x . 24962666

[66] Karamali M, Asemi Z, Ahmadi-Dastjerdi M, Esmaillzadeh A. Calcium plus vitamin D supplementation affects pregnancy outcomes in gestational diabetes: randomized, double-blind, placebo-controlled trial. Public Health Nutr. 2016;19(1):156–63. 10.1017/S1368980015000609 S1368980015000609 [pii]. . 25790761 PMC10271001

[67] Kanchan T, Machado M, Rao A, Krishan K, Garg AK. Enamel hypoplasia and its role in identification of individuals: A review of literature. Indian J Dent. 2015;6(2):99–102. 10.4103/0975-962X.155887 26097340 PMC4455163

