# Supplementary figures and images for: Prenatal exposure to gestational diabetes mellitus increases developmental defects in the enamel of offspring
Source: PLoS One. 2019 Feb 27;14(2):e0211771. doi: 10.1371/journal.pone.0211771 (PMC6392233; doi:10.1371/journal.pone.0211771)

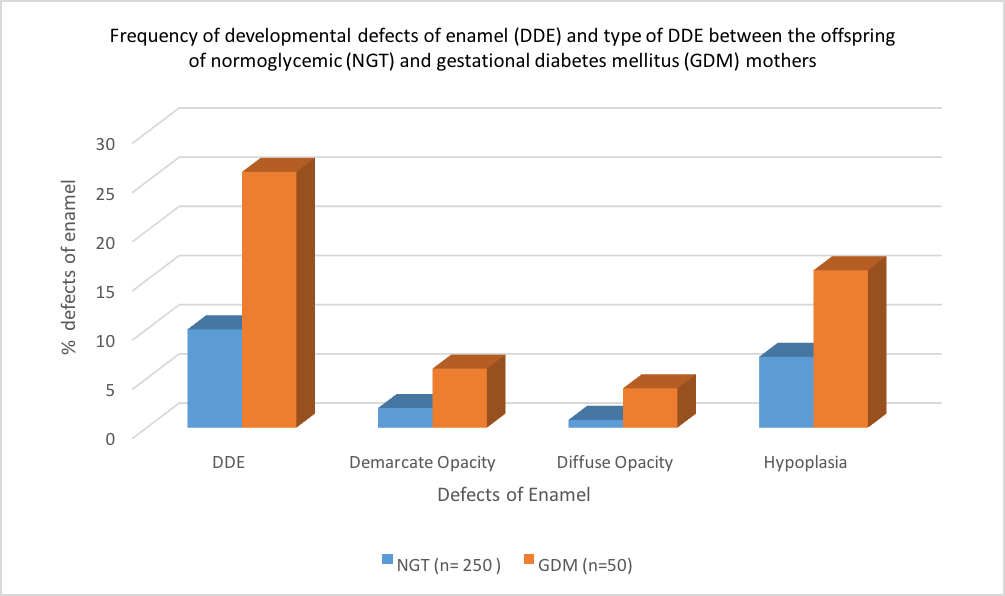

Supplement: S1 Fig — (PNG) [file pone.0211771.s001.png]

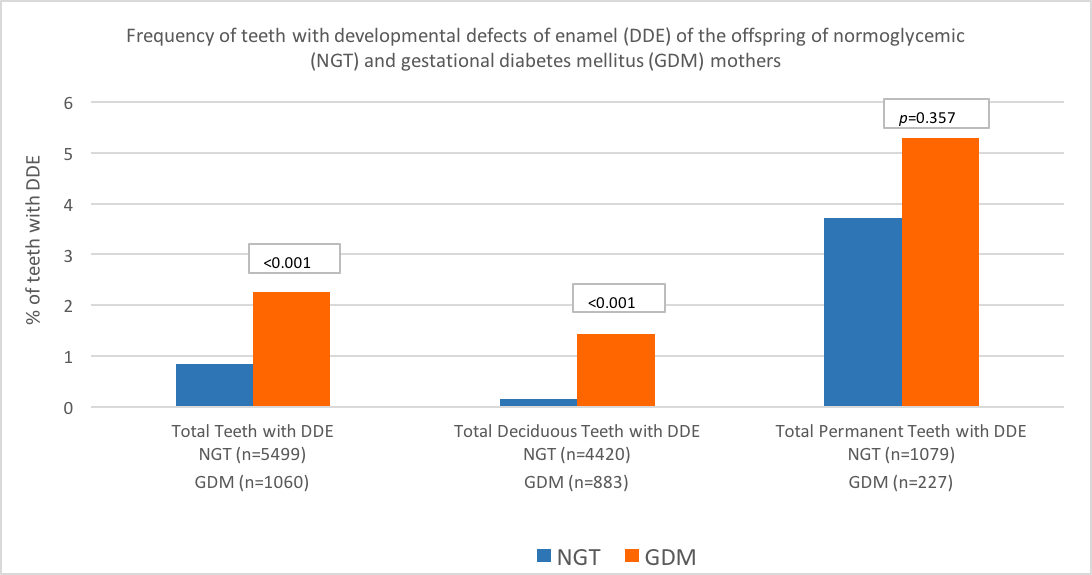

Supplement: S2 Fig — (PNG) [file pone.0211771.s002.png]
